# Large T Antigen-Specific Cytotoxic T Cells Protect Against Dendritic Cell Tumors through Perforin-Mediated Mechanisms Independent of CD4 T Cell Help

**DOI:** 10.3389/fimmu.2014.00338

**Published:** 2014-07-17

**Authors:** Anaïs Duval, Silvia A. Fuertes Marraco, Dominik Schwitter, Line Leuenberger, Hans Acha-Orbea

**Affiliations:** ^1^Department of Biochemistry, Center of Immunity and Infection Lausanne, University of Lausanne, Lausanne, Switzerland

**Keywords:** large T antigen, dendritic cell, CD8 T cell, CD4 T cells, perforin, tolerance

## Abstract

Our newly generated murine tumor dendritic cell (MuTuDC) lines, generated from tumors developing in transgenic mice expressing the simian virus 40 large T antigen (SV40LgT) and GFP under the DC specific promoter CD11c, reproduce the phenotypic and functional properties of splenic wild type CD8α^+^ conventional DCs. They have an immature phenotype with low co-stimulation molecule expression (CD40, CD70, CD80, and CD86) that is upregulated after activation with toll-like receptor ligands. We observed that after transfer into syngeneic C57BL/6 mice, MuTuDC lines were quickly rejected. Tumors grew efficiently in large T transgene-tolerant mice. To investigate the immune response toward the large T antigen that leads to rejection of the MuTuDC lines, they were genetically engineered by lentiviral transduction to express luciferase and tested for the induction of DC tumors after adoptive transfer in various gene deficient recipient mice. Here, we document that the MuTuDC line was rejected in C57BL/6 mice by a CD4 T cell help-independent, perforin-mediated CD8 T cell response to the SV40LgT without pre-activation or co-injection of adjuvants. Using depleting anti-CD8β antibodies, we were able to induce efficient tumor growth in C57BL/6 mice. These results are important for researchers who want to use the MuTuDC lines for *in vivo* studies.

## Introduction

The simian virus 40 large T antigen (SV40LgT) is a viral oncoprotein capable of transforming murine cells and recognized to induce tumorigenesis by binding to and inhibiting specific cellular tumor suppressor proteins such as p53 or retinoblastoma protein (pRb). However, immunocompetent C57BL/6 mice are protected against SV40LgT tumorogenesis after adoptive transfer. Indeed C57BL/6 mice are able to mount a specific MHC-I cytotoxic response to the SV40LgT oncogene through the recognition of four H-2^b^ restricted cytotoxic epitopes inside the large T antigen sequence (1, 2). Consequently, immunization of immunocompetent C56BL/6 mice with tumor cells expressing the SV40LgT oncogene results in a strong cytotoxic T lymphocytes (CTL) response leading to tumor rejection. Consequently, the CD8 T cell response was required for the rejection of the SV40 Large T tumors. This cytotoxic response can be mediated through various contact dependent pathways in the immunological synapse including Fas, tumor necrosis factor (TNF), TNF-related apoptosis-inducing ligand (TRAIL), or granule exocytosis.

We show here that the transfer of newly developed murine tumor dendritic cell (MuTuDC) lines derived from splenic tumors of transgenic CD11c:SV40LgT mice (3, 4) into immuno-deficient hosts (Rag2^−/−^) and transgene-tolerant mice (transgenic CD11c:SV40LgT) resulted in the development of splenic and hepatic DC tumors. In contrast, syngeneic immunocompetent C57BL/6 mice were resistant to the development of the MuTuDC tumor. Due to the lack of rejection in Rag2-deficient mice, we postulated that DC lines are immunogenic and are rejected in WT mice due to their ability to mount an immune response against the antigens inherent to the transgene of the MuTuDC lines, i.e., the SV40LgT oncogene and/or GFP.

As these cell lines are more and more utilized by different groups in research and it is often too cumbersome to breed all the recipient KO and transgenic mice to Rag deficiency or transgene tolerance, we wanted to characterize the rejection mechanisms to allow adoptive transfer into immune-competent mice. In order to investigate the mechanism of rejection of the MuTuDC lines, we lentivirally transduced the MuTuDC lines with luciferase as a reporter to follow tumor growth by non-invasive *in vivo* imaging. Using various recipient mice and depleting antibodies, we observed a striking tolerance to growth of MuTuDC lines in mice devoid of CD8^+^ T cells or perforin and conclude that MuTuDC lines were rejected from C57BL/6 mice via perforin-mediated lysis by CD8^+^ T cells. We additionally show that the rejection is CD4 T cell independent. The rejection was due to a fulminant immune CD8 T cell response against the large T antigen. This finding allows efficient transfer of MuTuDCs into immune-competent mice after CD8β depletion using monoclonal antibodies. Interestingly, despite the fact that immature DC usually induces tolerance, the cell line did not require activation to induce protective tumor immunity.

## Materials and Methods

### Mouse strains

C57BL/6 mice were purchased from Harlan laboratories. Rag2^−/−^, Ubi-GFP, pfn-deficient, CD3- deficient, generalized lymphoproliferative disease (Gld), CD11c-eGFP-DTR, and CD11c:SV40LgT mice were maintained under specific-pathogen free conditions in our animal facility. In each experiment, at least three mice of each strain were used and the animals were aged between 6 and 12 weeks. The experiments were performed in a conventional animal facility. The experiments were approved and controlled by the Swiss cantonal and federal veterinary authorities as well as by the local animal facility (Permission No. 2492).

### MuTuDC lines

MuTuDC lines, named for murine tumor, are derived from splenic tumors in transgenic CD11c:SV40LgT C57BL/6 mice (3). The derivation method is described in Ref. (4) and the MuTuDC lines display the phenotypic and functional features of the natural splenic CD8α^+^ conventional DC. MuTuDC1940 line was used for this study (4). MuTuDC lines were cultured in IMDM – Glutamax (GIBCO) supplemented with 8% of heat-inactivated fetal calf serum (FCS), 10 mM HEPES (GIBCO), 50 μM β-mercaptoethanol (GIBCO), 50 U/ml of penicillin, and 10 μg/ml streptomycin (GIBCO) at 37°C in 5% CO2 atmosphere. These cells were harvested in a non-enzymatic cell dissociation buffer (PBS 1×, 10 mM HEPES, and 5 mM EDTA). The number of cells was determined by using the Casy^®^ cell counter.

### Antibodies

Hybridoma cell line producing H35 was grown in IMDM culture medium supplemented with 2% of IgG-depleted FCS, 10 mM HEPES, and 50 μM β-mercaptoethanol at 37°C in 5% CO2 atmosphere. The anti-CD8β mAb was purified from cell culture supernatant of the H35 hybridoma on Protein G sepharose column (Amersham). Before immuno-staining, dead cells were excluded with the fixable viability dye eFluor506 (eBioscience). All antibodies used for flow cytometry experiments were diluted in an anti-mouse Fc receptor mAb purified from the supernatant of culture of the 2.4G2 hybridoma. Fluorochrome-conjugated monoclonal antibodies for flow cytometry were purchased from eBioscience: CD3-eFluor450 (eBio500A2), CD11c-PECy7 (N418), CD8α PerCpCy5.5 (53-6.7), CD62L Alexa Fluor700 (MEL-14), CD44 APC (IM7), and IFNγ PECy7 (XMG1.2) or BioLegend: CD19 PE (6D5) and CD4 APCCy7 (RM4-5). Analyses were performed with the FACSLSRII or FACSCanto machines (Becton Dickinson) using the FACSDiva software for the acquisition and the FlowJo software for data analyses.

### Generation of Luc-DC

The amplification of the *Photinus pyralis luciferase* gene was performed by PCR from the ISRE cis-reporter plasmid (Stratagene), kindly provided by Professor Jürg Tschopp’s group. Oligonucleotides used as PCR primers were synthesized according to the NCBI sequences: forward primer: 5′-GATCGGATCCGCCACCATGGAAGACGCCAAAAACAT-3′ and reverse primer: 5′-GATCGTCGACTCACAATTTGGACTTTCCGCCCT-3′. The amplification was carried out with 0.2 mM dNTP (Roche), 1 × expand high fidelity buffer (Roche), 0.35 units expand high fidelity polymerase (Roche), 4 mM MgCl2, 0.5 mM luciferase specific primers, and 0.1 ng ISRE cis-reporter plasmid. The first 10 cycles consist of 30 s at 94°C, 30 s at 56°C, and 70 s at 72°C with a loss of 1°C for the annealing step at each cycle, followed by 20 cycles of 30 s at 94°C, 30 s at 46°C, and 70 s at 72°C, followed by a final extension at 72°C for 10 min.

The luciferase PCR product and the (pWP-SIN-cPPT-WPRE)-CMV-IRES-GFP lentivirus vector were digested by *Bam*HI and *Sal*I (New England Biolabs). The purified insert was ligated into the prepared (pWP-SIN-cPPT-WPRE)-CMV-IRES-GFP lentivirus vector with 20 units of T4 DNA ligase (Rapid ligation kit, Promega). The transduction was done into the MuTuDC1940 line as previously described (4, 5).

### Detection of luciferase expression

The luciferase expression was checked by measuring the luciferase activity in Luc-DC by using the luciferase assay system kit (Promega) and the TD-20/20 Luminometer (Promega). The 2 × 10^6^ Luc-DC or parental MuTuDC lines were used for the experiment. The light emission was measured as relative light units (RLU). The luciferase activity in Luc-DC was also measured by using the Xenogen imaging system (Xenogen/Caliper life science, Platform of the Cellular Imaging Facility (CIF), University of Lausanne). From 1575 to 100,000 Luc-DC or parental MuTuDC lines were plated into a 96-well plate and the bioluminescence was monitored after addition of 0.15 mg/ml of d-luciferin and quantified as photons/s/cm^2^/sr.

### Adoptive transfer of the MuTuDC lines and bioluminescence imaging of the DC tumor development

The 5 × 10^6^ MuTuDC lines were injected by the intravenous (iv) route into the tail vein of mice. Clinical signs of tumor development were followed daily and mice were sacrificed when they showed reduced hematocrits. Upon transfer of 5 × 10^6^ Luc-DC cells, the DC tumor development was monitored after intraperitoneal (ip) injection of 1.5 mg/100 μl of d-luciferin (Promega, VivoGlo™ Luciferin) using the Xenogen imaging system. The images were analyzed using the LivingImage3.2 software and the luminescence was quantified as photons/s/cm^2^/sr.

### *In vivo* depletion of CD4^+^, CD8β^+^, and CD11c^+^ cells using mAb or DT treatment

Two days before and 3 days after Luc-DC injection, C57BL/6 mice were injected ip with 100 μg of anti-CD8β clone H35 mAb or 750 μg GK1.5 ascites fluid. The *in vivo* depletion of lymphocyte population was confirmed by flow cytometry by examining blood cell population and was above 97%. CD11c-eGFP-DTR mice and littermates were treated by ip injection with 4 ng of DT per gram of weight 1 day before the iv injection of Luc-DC. The *in vivo* depletion of CD11c^+^ cells was analyzed by flow cytometry in the spleen 1 day after the DT injection.

### *Ex vivo* restimulation of splenocytes

When tumor-bearing mice displayed clinical symptoms, all Luc-DC injected mice were sacrificed (about 15–20 days following Luc-DC transfer according to the experiment) and spleens were digested by incubating organs for 20 min at 25°C in 1 mg/ml collagenase D (Roche), 40 μg/ml DNAse I (Roche) in RPMI 1640 (GIBCO) supplemented with 50 μg/ml gentamycin, 5% FCS, and 1 mM HEPES. Cells were washed with complemented RPMI medium and 5 μg/ml DNAse I and mechanically disrupted on a 40-μm nylon cell strainer. Erythrocytes were lysed with the red blood cell lysis buffer (0.01 M KHCO_3_ + 0.155 M NH_4_Cl + 0.1 mM EDTA, pH 7.5) and cells were counted using the Casy^®^ cell counter. The 0.3 × 10^6^ splenocytes were restimulated or not with 1 × 10^4^ parental MuTuDC line previously seeded. In other experiments, splenocytes were restimulated or not with 0.3 × 10^6^ C57BL/6 splenocytes and 8.1 μg of recombinant SV40LgT protein (ChimerX). After 4 days, T cells were restimulated with 10 ng/ml of PMA (Sigma), 500 ng/ml of ionomycin (Calbiochem), and 10 μg/ml of brefeldin A (Sigma) for 4 h at 37°C and the activation and differentiation state of T cells were assessed by flow cytometry. Supernatants were assessed for IFNγ secretion by ELISA by using kits from eBioscience according to the provider’s instructions.

### Statistical analyses

When indicated, statistical analyses were performed with Prism software. Differences between two groups were assessed by a two-ways ANOVA test followed by Bonferroni *post hoc* test (*p < 0.05; **p < 0.005; ***p < 0.001).

## Results

### C57BL/6 mice are resistant to syngeneic MuTuDC tumor development by an adaptive response

**Figure 1:**
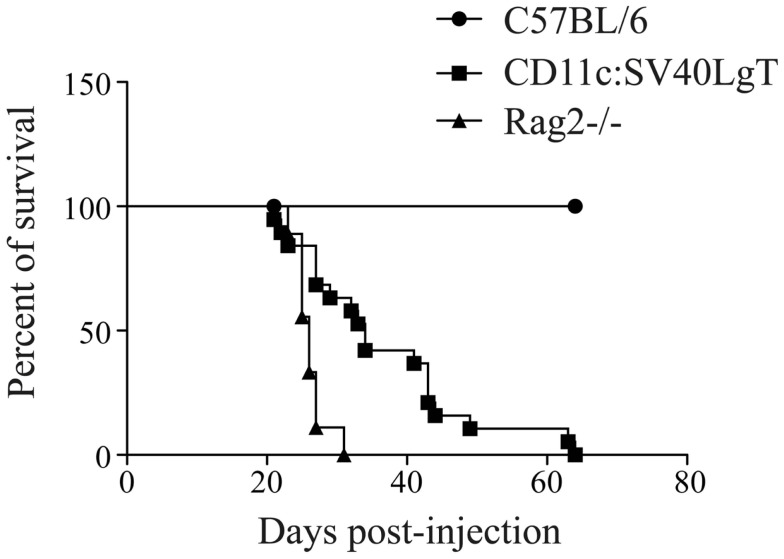
**MuTu tumors developed in tolerant CD11c:SV40LgT and Rag-deficient mice but not in immunocompetent C57BL/6 mice**. Five × 10^6^ MuTuDC lines were transferred by iv route into C57BL/6 (*n* = 19), CD11c:SV40LgT (*n* = 19), or Rag2^−/−^ mice (*n* = 9). The percent of survival was displayed according to the day post-injection.

Upon adoptive transfer of the MuTuDC lines, C57BL/6 mice did not develop DC tumors (Figure 1). The mice were sacrificed 200 days post-injection and no signs of splenomegaly or hepatosplenomegaly were observed. Conversely, upon transfer of the MuTuDC lines into immunocompetent transgene-tolerant CD11c:SV40LgT mice, hepatic and splenic tumors developed readily (Figure 1). Two CD11c:SV40LgT mouse lines exist, one with fast-developing tumors (line 13, tumors at 3.5 months, from which most MuTuDC lines were originally derived), and the second one with slow-developing tumors (line 2, tumors at 12 months) (3). Line 2 mice were used in the transfer experiment to avoid growth of endogenous tumors and the ability to distinguish endogenous from transferred tumors due to different eGFP expression levels distinguishable by flow cytometry. CD11c:SV40LgT line 2 mice developed splenomegaly and hepatomegaly in average 1 month after the MuTuDC line transfer, instead of after 12 months without transfer. Furthermore, after transfer of the MuTuDC lines into Rag2^−/−^ mice, lacking T and B cells due to the absence of the recombination activating gene, the mice developed tumors in the liver and the spleen in about 1 month (Figure 1). These Results indicated that the induction of an anti-tumor immunity in C57BL/6 mice is inherent to tumor antigens expressed by the MuTuDC lines and that the tumor protection is mediated by a T and/or B cell response eliminating the DC tumors in C57BL/6 mice.

### Generation of luciferase-expressing MuTuDC line

**Figure 2:**
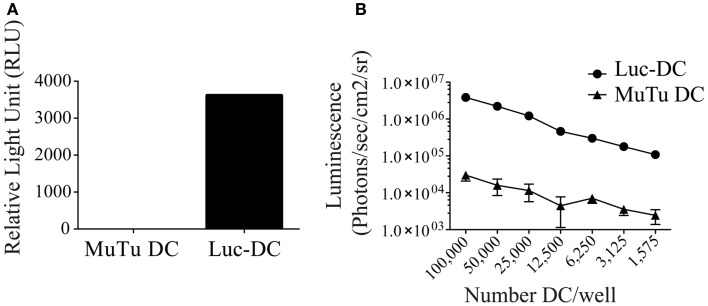
**Luc-DC efficiently expressed the luciferase**. MuTuDC lines were transduced with the lentivirus vector containing the gene encoding the *Photinus pyralis luciferase* and the luciferase activity was controlled *in vitro*. **(A)** The 2 × 10^6^ cells were lysed and the bioluminescence was quantified by using a luminometer after adding of the d-luciferin. The light emission was measured as relative light units (RLU). **(B)** Different numbers of cells were plated in triplicate in a 96-well plate and the bioluminescence was monitored and quantified as photons/s/cm^2^/sr after adding 0.15 mg/ml of d-luciferin by using the Xenogen imaging system. Data are presented as mean ± SD.

To investigate the MuTuDC line clearance mechanism in C57BL/6 mice, we generated a luciferase-expressing MuTuDC line by lentiviral transduction (named Luc-DC hereafter). The efficacy of the lentiviral transduction was determined by the measure of the bioluminescence activity of Luc-DC after 1 week (Figure 2A) and was stable for up to 10 passages in culture (data not shown). The emission of bioluminescence was cell-number dependent (Figure 2B). Consequently, Luc-DC efficiently expressed the luciferase and were used to follow tumor development by *in vivo* imaging in living animals.

### DC tumor rejection in syngeneic mice is not compromised by luciferase expression

**Figure 3:**
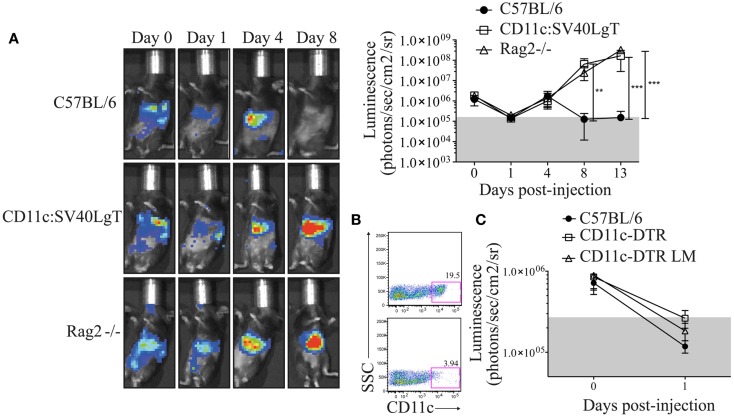
**C57BL/6 mice are resistant to Luc-DC line tumor and the depletion of endogenous CD11c^+^ cells did not provide a niche for the initial expansion of MuTuDC line**. Five × 10^6^ Luc-DC were transferred by iv route and the DC tumor growth was followed over time by bioluminescence measurement using the Xenogen imaging system after ip injection of 1.5 mg of d-luciferin. The bioluminescence was quantified as photons/s/cm^2^/sr. **(A)** The tumor development was followed in C57BL/6 (*n* = 9), CD11c:SV40LgT (*n* = 6), and Rag2^-/-^ (*n* = 6) mice at days 0, 1, 4, 8, and 13 following the transfer of Luc-DC. One representative mouse is shown. **(B)** CD11c-eGFP-DTR littermates (upper panel) and CD11c-eGFP-DTR mice (bottom panel) were injected with DT and the efficiency of the depletion of CD11c^+^ cells was checked in the spleen 24 h later by flow cytometry (gated on single CD3^-^ CD19^-^ cells). **(C)** Luc-DC were transferred into C57BL/6 (*n* = 7), DT-treated CD11c-eGFP-DTR (*n* = 8), and DT-treated CD11c-eGFP-DTR littermates (*n* = 7) mice. CD11c-eGFP-DTR mice were treated with the DT, 1 day prior the transfer of the Luc-DC. The tumor growth was assessed just after the transfer and at day 1. Data are presented as mean ± SD. The *p* values were determined by a two-way ANOVA with Bonferroni post-test.

In order to determine whether the luciferase expression did not change the behavior of the MuTuDC lines *in vivo*, Luc-DC were transferred into syngeneic C57BL/6, CD11c:SV40LgT, and Rag2^-/-^ mice and the Luc-DC expansion was followed by measuring the bioluminescence signal. After iv injection, cells circulated and primarily localized to the lung, spleen, and liver. The bioluminescence signal decreased after 1 day and thereafter surviving Luc-DC started to expand mainly in the liver and the spleen. Tumors comparably grew in CD11c:SV40LgT and Rag2^-/-^ recipients. *In vivo* imaging showed the implantation of the DC tumors in the spleen and the liver 8 days after Luc-DC transfer (Figure 3A). Conversely, after 8 days, no more luciferase activity was detected in C57BL/6 mice due to elimination of Luc-DC (Figure 3A). Consequently, Luc-DC, similar to the parental MuTuDC lines, failed to expand and develop tumors in C57BL/6 mice but induced tumors in immuno-deficient and transgene-tolerant mice. Consequently, the luciferase expression did not alter the MuTuDC lines and therefore Luc-DC nicely recapitulate the observations using the WT MuTuDC lines, providing an imaging method to follow tumor progression *in vivo*.

### The initial loss of MuTuDC lines at day 1 was not altered by endogenous DC depletion

As observed in Figure 3A, the *in vivo* Luc-DC number declined 1 day following transfer into WT, Rag-deficient, and transgene-tolerant mice. To investigate whether this reduced signal was due to the absence of an available niche for the initial proliferation of transferred cells, we used CD11c-eGFP-DTR recipient mice that express the diphtheria toxin receptor (DTR) under the CD11c promoter (6). CD11c^+^ cells were efficiently depleted in diphtheria toxin (DT)-treated CD11c-eGFP-DTR mice (Figure 3B) and this depletion could provide the space and resources required for the expansion of the transferred DC lines. One day following Luc-DC injection, the bioluminescence signal was equivalent in DT-treated CD11c-eGFP-DTR, DT-treated CD11c-eGFP-DTR littermates, and C57BL/6 mice (Figure 3C), therefore the depletion of CD11c^+^ cells did not prevent the decrease in the bioluminescence signal observed at day 1 following Luc-DC transfer. Consequently, the decline of Luc-DC numbers could not be explained by the lack of a niche required for the initial expansion.

### MuTuDC lines were cleared in C57BL/6 mice by perforin-producing CTL independent on CD4 T cell help

**Figure 4:**
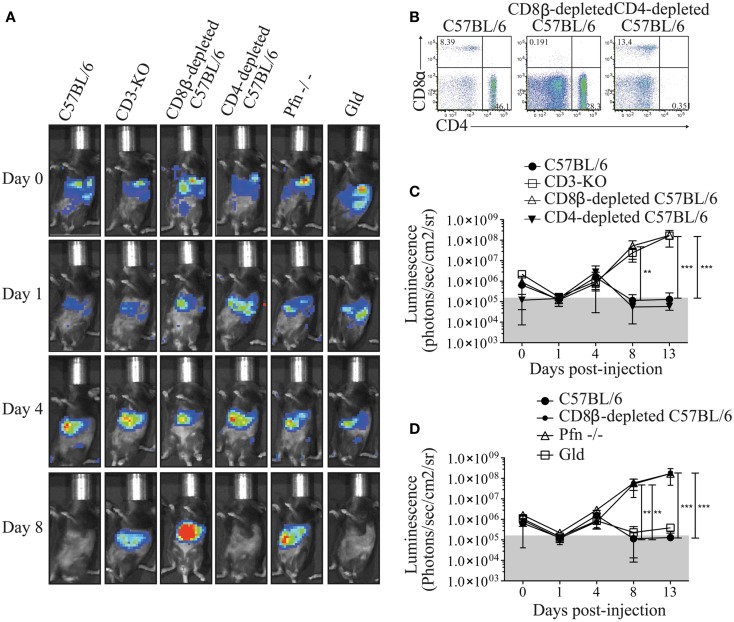
**Perforin-producing CD8^+^ T cells protect C57BL/6 mice from DC tumor development**. **(A)** The tumor growth was detected by monitoring the bioluminescence after iv injection of 5 × 10^6^ Luc-DC into C57BL/6 (*n* = 12), CD3-KO (*n* = 3), CD8β-depleted C57BL/6 (*n* = 9), CD4-depleted C57BL/6 (*n* = 6), Pfn^−/−^ (*n* = 6), and Gld (*n* = 6) recipient mice at different time points. The bioluminescence was assessed by using the Xenogen imaging system after ip injection of 1.5 mg of d-luciferin at days 0, 1, 4, 8, and 13 after Luc-DC injection. **(B)** Two days before and 3 days after Luc-DC injection, 100 μg of CD8β- or CD4-depleting antibody were administrated into C57BL/6 mice by ip injections. The effective depletion of CD8^+^ and CD4^+^ cells was checked by flow cytometry in the blood 1 day after the last injection (gated on single live cells). **(C,D)** The bioluminescence measured in the various mouse strains was quantified as photons/s/cm^2^/sr. Data are presented as mean ± SD. The *p* values were determined by a two-way ANOVA with Bonferroni post-test.

In order to investigate the mechanism by which MuTuDC lines are cleared from C57BL/6 mice, Luc-DC were injected into CD3-KO mice. As observed in Figure 4A,C, Luc-DC formed tumors in CD3-deficient mice. To further explore the T cell-mediated rejection, tumor development was assessed in CD8β- and CD4-depleted C57BL/6 mice after Luc-DC challenge. The effective depletion was of 97% for CD8^+^ T cells and superior to 98% for CD4^+^ T cells (Figure 4B). Eight days after challenge, Luc-DC were cleared from WT and CD4-depleted C57BL/6 mice, whereas CD8β-depleted mice failed to eliminate the MuTuDC lines leading to tumor outgrowth (Figure 4A**,C**). Therefore, the elimination of Luc-DC in C57BL/6 mice is dependent on CD8^+^ T cell-dependent killing and does not require CD4 T cell help. Furthermore, Luc-DC tumors grew progressively in perforin (Pfn)-deficient mice but not in FasL-deficient mice (Gld) upon transfer (Figure 4A**,D**). Consequently, the loss of perforin-dependent cytolytic function breaks the immune resistance and allows tumor outgrowth.

### Only CD8^+^ T cells from tumor-rejecting mice responded strongly after *in vitro* restimulation

**Figure 5:**
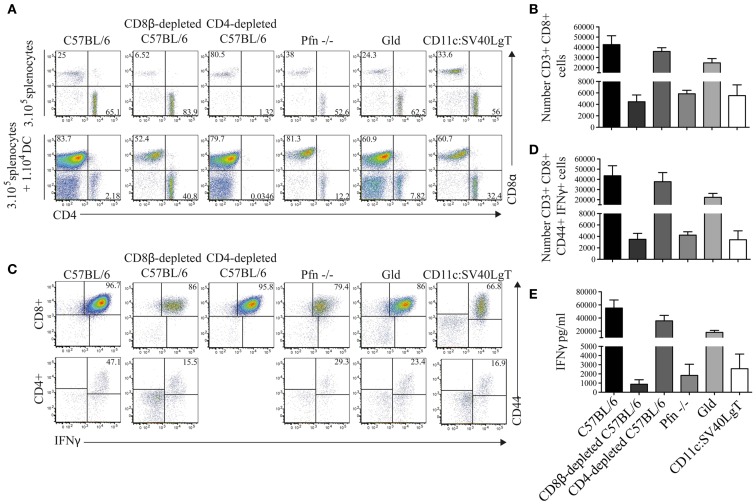
**CD8^+^ T cells from tumor-free mice highly proliferated after *ex vivo* restimulation with the parental MuTuDC lines**. **(A,B)** Mice were sacrificed 14 days after Luc-DC transfer and 3 × 10^5^ splenocytes were *ex vivo* restimulated with 1 × 10^4^ MuTuDC lines (upper panel) or left untreated in 96-well plate (lower panel). After 4 days, the CD8 and CD4 T cell response was analyzed by flow cytometry **(A)** and the numbers of CD4^+^ and CD8^+^ T cells after restimulation with MuTuDC lines were quantified after gating on single CD3^+^ live cells **(B)**. **(C,D)** The expression of the activation marker CD44 and the production of IFNγ were analyzed, after restimulation with the MuTuDC lines for 4 days, among CD8^+^ CD4^−^ cells (upper panel) and CD4^+^ CD8^−^ cells (lower panel) after gating on single CD3^+^ live cells **(C)** and the number of CD3^+^ CD8^+^ CD4^−^ CD44^+^ IFNγ^+^ cells was quantified **(D)**. **(E)** The secretion of IFNγ was assessed by ELISA in the supernatant of culture after restimulation with the MuTuDC lines for 4 days. Mean ± SD is shown. Results are representative of two independent experiments.

To further analyze the CTL response, splenocytes from Luc-DC-injected mice were *ex vivo* restimulated with the parental MuTuDC lines (not expressing Luciferase). After 4 days of co-culture of splenocytes with DC lines, we observed a CD8^+^ T cell response that was markedly higher in splenocytes from the C57BL/6, CD4-depleted, and FasL-deficient mice than in splenocytes from the tumor-bearing CD8β-depleted, Pfn-deficient, and CD11c:SV40LgT hosts (Figure 5A**,B**). These CD8^+^ T cells expressed the activation marker CD44 and produced IFNγ (Figure 5C**–E**). Conversely, very small numbers of CD4^+^ were detected and only weakly upregulated CD44 and IFNγ (Figure 5C), confirming the CTL-mediated response to the MuTuDC lines.

## Discussion

MuTuDC lines are immunogenic and are quickly rejected upon transfer into H-2^b^ C57BL/6 mice by a perforin-mediated CD4 T cell independent of CD8^+^ T cell response. DC tumor development in Rag-deficient mice suggests that NK cells were not sufficient to induce MuTuDC line rejection and the presence of tumor development in perforin-deficient mice demonstrated that other known mechanisms of cytotoxicity (7) are not important for tumor clearance and of minor importance.

We assume here that this CD8 T cell response is mediated against the transgenic SV40LgT and not the GFP endogenous to the MuTuDC lines. Indeed Ubi-GFP mice were able to eliminate the MuTuDC lines. The transfer of the MuTuDC lines into GFP-expressing mice did not induce DC tumor outgrowth meaning that SV40LgT antigen alone was sufficient to induce the protective anti-tumor immunity. In addition, using recombinant large T protein, we were able to show reactivity of splenocytes of MuTuDC immunized mice to this antigen *in vitro* (Figure S1 in Supplementary Material). The anti-tumor response could not be attributed to the luciferase expressed by the injected Luc-DC since first, luciferase has been shown to be poorly immunogenic (8, 9); second, Luc-DC induce tumors in large T and GFP-tolerant mice; and third, because CD8^+^ T cells isolated from tumor-free Luc-DC-injected C57BL/6 mice highly proliferated *in vitro* in response to the parental MuTuDC lines devoid of luciferase expression. We also can exclude that the MuTuDC lines reveal new antigens in C57BL/6 recipients due to modifications acquired during the *in vitro* immortalization since tumors easily grew in mice tolerant to CD11c:SV40LgT.

The decline of Luc-DC numbers during the first day after transfer could be attributed to the death of injected cells as it was not reverted by providing niche spaces. Indeed several studies showed that radioactive-labeled tumor cells died by apoptosis in the lung during the 24 h following the administration into the blood circulation (10, 11). It is coherent with the localization of Luc-DC in the lung in the hours following the injection.

It is intriguing that the transfer of the MuTuDC lines into C57BL/6 mice elicited an unhelped strong anti-tumor CD8 T cell response. DC that were not pre-activated by TLR ligands and display the same or lower level of activation markers (co-stimulation molecules and MHC-II) as freshly isolated splenic DC (3, 4) did not induce tolerance but rather immunity. Furthermore, no “spontaneous” activation of MuTuDC lines was observed during the culture and preparation of the cells as already observed for *ex vivo* isolated DC (12). Consequently, it is contrary to the concept in which DC have to be previously treated with maturation stimuli to induce an efficient T cell response (13–15). Thereby, a high immunogenicity or a transfer of a high number of MuTuDC lines allows counteracting the requirement of a previous maturation to induce immunity. Moreover, we demonstrated that the rejection of MuTuDC lines was mediated by an unhelped CD8 T cell response. However, in the literature, a protective anti-tumor CD8 immunity is associated with the CD4 T cell help (16) and thus the requirement of CD4 T cell helps to induce the CD8 T cell response to tumors that could be substituted by the high immunogenicity and frequency of MuTuDC lines.

After *ex vivo* restimulation, we observed a strong CD8 T cell response in tumor-free mice whereas the CD4 T cell response was low. In tumor-bearing mice, an anti-tumor CD8 T cell response was observed *ex vivo* but lower than the one observed in tumor-free hosts. We observed the following hierarchy in immune response in tumor-bearing mice: the CD8 T cell response was superior in CD8β-depleted mice and equivalent in pfn-deficient and SV40LgT and GFP-tolerant mice. In CD8β-depleted mice, we speculate that newly developed CTL were rendered tolerant *in vivo* and therefore were unable to control tumor growth as already described in a sporadic tumor model. Tumor cells were immunogenic and induced tolerance to a cellular anti-tumor immunity, not able to protect mice from tumor development (17). The lower tumor-specific proliferative response of pfn-deficient CTL was surprising as several studies showed that perforin-deficient mice exhibited an exaggerated CTL immune response upon infections (18–20). The mechanisms of partial tolerance induction have to be investigated but we speculate that in tumor-bearing mice, perforin-deficient CD8^+^ T cells were exhausted due to tumor environment. However, we could not exclude that CD8^+^ T cells in perforin-deficient mice were not primed after MuTuDC line injection. Indeed several studies showed that the priming of T cells in DC vaccination relied on the transfer of antigens to endogenous DC that capture and present antigens from injected/killed DC to CD8^+^ T cells (21, 22). The deficiency in perforin could avoid the killing of injected cells and therefore render unable the capture of antigens by endogenous APC. This hypothesis is supported by the absence of proliferation of ova-specific CD8^+^ T cells observed after injection of ova-loaded MuTuDC lines in Kb^−/−^, β2m^−/−^, Batf3^−/−^, and CD11c-depleted mice whereas OT-I T cells nicely proliferated in C57BL/6 mice (data not shown). Surprisingly, CD8^+^ T cells from transgene-tolerant mice also responded to MuTu DC lines upon rechallenge whereas tolerance should be complete due to antigen expression by all thymic DC. However, it could argue for the presence of an antigen acquired by the MuTuDC lines during the *in vitro* immortalization process and which elicits the CD8 T cell response. This argument is counteracted by the restimulation of the CD8 T cell response with recombinant large T antigen. These findings demonstrate that SV40-transformed non-activated MuTuDC lines are immunogenic and thereby elicit a strong CD8 T cell response in syngeneic mice. As these DC lines are now distributed worldwide, we wanted to investigate the behavior of these cells in their syngeneic mouse strain because these DC lines are expected to serve as a tool to help design of new strategies for immunotherapies for the treatment of cancer and autoimmune disorders that then can be confirmed using classical DC vaccination.

## Conflict of Interest Statement

The authors declare that the research was conducted in the absence of any commercial or financial relationships that could be construed as a potential conflict of interest.

## Supplementary Material

The Supplementary Material for this article can be found online at http://www.frontiersin.org/Journal/10.3389/fimmu.2014.00338/abstract
