# Supplementary figures and images for: Large T Antigen-Specific Cytotoxic T Cells Protect Against Dendritic Cell Tumors through Perforin-Mediated Mechanisms Independent of CD4 T Cell Help
Source: Front Immunol. 2014 Jul 17;5:338. doi: 10.3389/fimmu.2014.00338 (PMC4101877; doi:10.3389/fimmu.2014.00338)

Figure S1

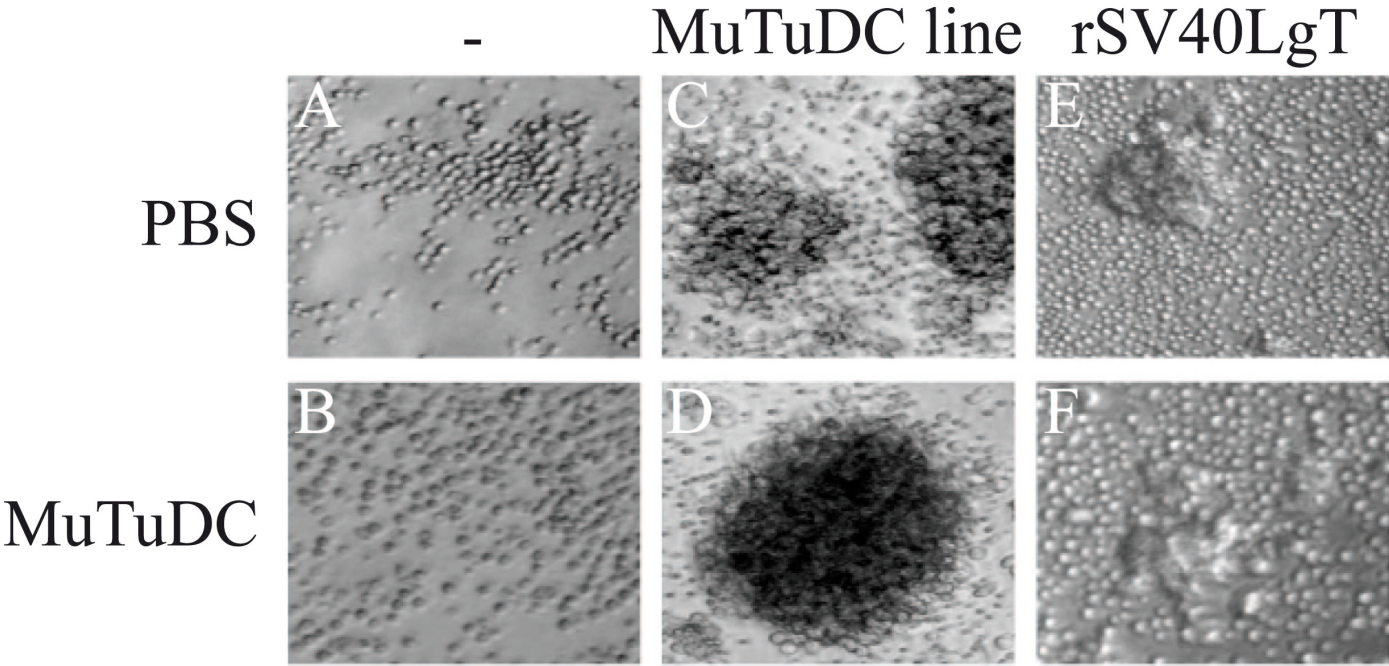

Supplement: Supplementary file 1 [file Presentation_1.PDF]
