# Supplementary material for: Large T Antigen-Specific Cytotoxic T Cells Protect Against Dendritic Cell Tumors through Perforin-Mediated Mechanisms Independent of CD4 T Cell Help
Source: Front Immunol. 2014 Jul 17;5:338. doi: 10.3389/fimmu.2014.00338 (PMC4101877; doi:10.3389/fimmu.2014.00338)
Supplement: Supplementary file 2 [file Data_Sheet_1.DOCX]

***Supplementary Material***

Large T antigen-specific cytotoxic T cells protect against dendritic cell tumors through perforin-mediated mechanisms independent of CD4 T cell help

**Anaïs Duval1,#, Silvia Fuertes-Marraco1.##, Dominik Schwitter1, Line Leuenberger1.###, Hans Acha-Orbea1***

^1^Department of Biochemistry, Center of immunity and infection Lausanne, University of Lausanne, Epalinges, Switzerland

#Present address: Institute of pharmacology and Structural Biology, Centre National de la Recherche Scientifique, University Paul Sabatier, University of Toulouse, Toulouse, France

##Present address: Clinical Tumor Biology and Immunotherapy Unit, Ludwig Institute for Cancer Research of the University of Lausanne, Lausanne, Switzerland

###Present address: Division of Immunology and Allergy, Lausanne University Hospital (CHUV), Lausanne, Switzerland

* **Correspondence:** Hans Acha-Orbea, Department of Biochemistry, Center of immunity and infection Lausanne, University of Lausanne, Chemin des Boveresses 155, CH-1066 Epalinges, Switzerland.

Email: hans.acha-orbea@unil.ch

## Suplementary Figures

**Supplementary Figure 1.** **Splenocytes from MuTuDC line-transferred C57BL/6 respond to the stimulation with the recombinant SV40LgT protein.**

Mice were sacrificed 21 days after PBS (upper panel) or MuTuDC line injection (lower panel) and 3.10^5^ splenocytes were *ex vivo* restimulated with 1.10^4^ MuTuDC line cells (panels C and D) or with 3.10^5^ splenocytes from naïve WT mice and 8.1 µg recombinant SV40LgT (panels E and F) or left untreated (panels A and B). Pictures were taken with a light microscopy.
